# Different applications of isosbestic points, normalized spectra and dual wavelength as powerful tools for resolution of multicomponent mixtures with severely overlapping spectra

**DOI:** 10.1186/s13065-017-0270-8

**Published:** 2017-05-25

**Authors:** Ekram H. Mohamed, Hayam M. Lotfy, Maha A. Hegazy, Shereen Mowaka

**Affiliations:** 1 Pharmaceutical Analytical Chemistry Department, Faculty of Pharmacy, The British University in Egypt, El-Sherouk City, 11837 Egypt; 2 Pharmaceutical Analytical Chemistry Department, Faculty of Pharmacy, Cairo University, Kasr El-Aini Street, Cairo, 11562 Egypt; 3 Pharmaceutical Chemistry Department, Faculty of Pharmaceutical Science & Pharmaceutical Industries, Future University, Cairo, 12311 Egypt; 4 Pharmaceutical Analytical Chemistry Department, Faculty of Pharmacy, Helwan University, Ein Helwan, Cairo, 11795 Egypt

**Keywords:** Derivative transformation, Advanced ratio difference, Induced ratio difference normalized spectra, Isosbestic point, Dual wave length

## Abstract

**Background:**

Analysis of complex mixture containing three or more components represented a challenge for analysts. New smart spectrophotometric methods have been recently evolved with no limitation. A study of different novel and smart spectrophotometric techniques for resolution of severely overlapping spectra were presented in this work utilizing isosbestic points present in different absorption spectra, normalized spectra as a divisor and dual wavelengths. A quaternary mixture of drotaverine (DRO), caffeine (CAF), paracetamol (PCT) and para-aminophenol (PAP) was taken as an example for application of the proposed techniques without any separation steps. The adopted techniques adopted of successive and progressive steps manipulating zero /or ratio /or derivative spectra. The proposed techniques includes eight novel and simple methods namely direct spectrophotometry after applying derivative transformation (DT) via multiplying by a decoding spectrum, spectrum subtraction (SS), advanced absorbance subtraction (AAS), advanced amplitude modulation (AAM), simultaneous derivative ratio (S^1^DD), advanced ratio difference (ARD), induced ratio difference (IRD) and finally double divisor–ratio difference-dual wavelength (DD-RD-DW) methods.

**Results:**

The proposed methods were assessed by analyzing synthetic mixtures of the studied drugs. They were also successfully applied to commercial pharmaceutical formulations without interference from other dosage form additives. The methods were validated according to the ICH guidelines, accuracy, precision, repeatability, were found to be within the acceptable limits.

**Conclusion:**

The proposed procedures are accurate, simple and reproducible and yet economic. They are also sensitive and selective and could be used for routine analysis of complex most of the binary, ternary and quaternary mixtures and even more complex mixtures.

## Background

Drotaverine (DRO) hydrochloride, 1-[(3,4-Diethoxy phenyl)methylene]-6,7-diethoxy-1,2,3,4-tetrahydroisoquinoline hydrochloride [1, 2] is non-anticholinergic antispasmodic drug.

Caffeine (CAF) 1,3,7-Trimethylpurine-2,6-Dione, is an adenosine receptor antagonist and adenosine 3′,5′cyclic monophosphate (cAMP) phosphodiesterase inhibitor, thus levels of cAMP increase in cells following treatment with caffeine [2, 3].

Paracetamol (PCT) *N*-(4-hydroxyphenyl) acetamide, also known as acetaminophen PAR is widely used as analgesic and antipyretic for the relief of fever, headaches and minor pains. It is a major ingredient in numerous cold and flu remedies [4, 5].

**Fig. 1:**
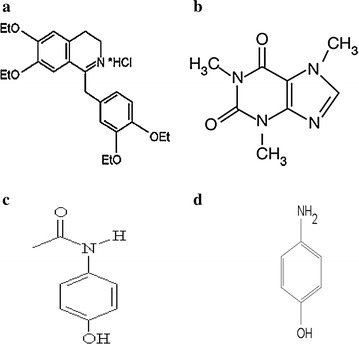
Structural formulae for **a** drotaverine, **b** caffeine, **c** paracetamol, **d** para-aminophenol

Para-aminophenol (PAP), is the primary impurity of PCT, it occurs in PCT pharmaceutical preparations as a consequence of both synthesis and degradation during storage [6, 7]. The quantity of PAP must be strictly controlled as it is reported to have nephrotoxic and teratogenic effects [7]. The structures of the studied drugs are presented in Fig. 1.

The analysis of mixtures containing DRO, CAF and PCT was described in few analytical reports. These reports proposed spectrophotometric [8, 9], TLC [9] and high performance liquid chromatography (HPLC) [8, 10, 11].

While literature survey reveals that no methods have been reported for the simultaneous determination of the four components under study.

The aim of this work was to develop novel spectrophotometric methods based on smart original mathematical techniques for resolving the quaternary mixture of DRO, CAF, PCT and PAP with spectral interfering problems.

## Theoretical background

Derivative transformation [12], spectrum subtraction [13], amplitude factor [14], advanced absorbance subtraction method (AAS) [15], advanced amplitude modulation method (AAM) [15] and simultaneous derivative ratio (S^1^DD) [16] are well developed method that were successfully adopted for resolution of overlapped spectra of binary mixtures.

For simultaneous determination of ternary mixtures two novel methods were newly proposed namely ratio difference-isosbestic points (RD-ISO) and induced ratio difference (IRD).

Ratio difference-isosbestic points (RD-ISO) is considered as an extension to ratio difference method [17]. The method requires the presence of two isosbestic points (λ_iso1_ and λ_iso2_) between two drugs for its successful application as discussed briefly.

If a ternary mixture X, Y and Z where (X and Y) shows two isoabsorptive points, Z can be determined by dividing the spectrum of the ternary mixture by normalized spectrum of X′.

The ratio spectra obtained using X′ as a divisor generated a constant value of its concentration along the whole spectra.

Suppose the amplitudes of the ratio spectra of the ternary mixture at the two selected wavelength (λ_iso1_ and λ_iso2_ between X and Y) are P_1_ and P_2_, respectively, then;

1 $${\text{P}}_{ 1} = \left[ {{\text{C}}_{\text{x}} } \right]\; + \;\left[ {{\text{C}}_{\text{Y}} } \right]\; + \;\left[ {{\text{a}}_{\text{z1}} {\text{C}}_{\text{z}} } \right]/{\text{a}}_{\text{x}}$$

2 $${\text{P}}_{ 2} = \;\left[ {{\text{C}}_{\text{x}} } \right]\; + \;\left[ {{\text{C}}_{\text{Y}} } \right]\; + \;\left[ {{\text{a}}_{\text{z2}} {\text{ C}}_{\text{z}} } \right]/{\text{a}}_{\text{x}}$$

By subtraction

3 $${\text{P}}_{ 1} - {\text{P}}_{ 2} = \left( {\frac{{a_{z} C_{z} }}{{a_{x} }}} \right)1 - \left( {\frac{{a_{z} C_{z} }}{{a_{x} }}} \right)2$$

The concentration of Z is calculated using the regression equation representing the linear correlation between the differences of ratio spectra amplitudes at the two selected wavelengths to the corresponding concentrations of drug (Z).

While IRD method is a combination between induced dualwavelength [18] and amplitude modulation theory. All what it need is the extension of one of the three drugs over the other two as summarized briefly.

The ratio spectra obtained using the normalized spectrum of the more extended component Z′ as a divisor generated a constant value of its concentration along the whole spectra that can be measured from the extended region parallel to the X axis.

The constant value of Z was then subtracted from the total ratio spectrum of the ternary mixture to obtain the ratio spectra of the other two components X and Y.

For determination of X, two wave lengths were selected in the ratio spectra of the resolved binary mixture. A remarkable amplitude difference between the two selected wavelengths in the ratio spectra of pure X should be present. To cancel the contribution of Y at the two selected wavelengths upon obtaining the ratio difference, the equality factor of pure ratio spectra of Y at these wavelengths (F_Y_) is calculated.

4 $${\text{Pm}}_{ 1} = {\text{P}}_{\text{X1}} \; + \;{\text{P}}_{\text{Y1}} \quad {\text{at }}\lambda_{ 1}$$

5 $${\text{Pm}}_{ 2} = {\text{P}}_{\text{X2}} \; + \;{\text{P}}_{\text{Y2}} \quad {\text{at }}\lambda_{ 2}$$

$${\text{F}}_{\text{Y}} = {\text{P}}_{\text{Y1}} /{\text{P}}_{\text{Y2}}$$

$$\therefore {\text{P}}_{\text{Y1}} = {\text{F}}_{\text{Y}} {\text{P}}_{\text{Y2}}$$

By substituting in Eq. (4)

6 $${\text{Pm}}_{ 1} = {\text{P}}_{\text{X1}} \; + \;{\text{F}}_{\text{Y}} {\text{P}}_{\text{Y2}}$$

By multiply Eq. (5) by F_Y_

7 $${\text{F}}_{\text{Y}} {\text{Pm}}_{ 2} = {\text{F}}_{\text{Y}} {\text{P}}_{\text{X2}} \; + \;{\text{F}}_{\text{Y}} {\text{P}}_{\text{Y2}}$$

And by calculating the difference, Eqs. (6, 7), F_Y_ P_Y2_ will be cancelled:

8 $$\Delta {\text{P }}\left( {{\text{Pm}}_{ 1} \;{-}\;{\text{F}}_{\text{Y}} {\text{Pm}}_{ 2} } \right) = {\text{A}}_{\text{X1}} \; - \;{\text{F}}_{\text{Y}} {\text{A}}_{\text{X2}}$$

Equation (8) indicated that the amplitude difference of the ratio spectra of the resolved binary mixture X, Y is dependent only on X and independent on Y.

The concentration of Y is calculated using the same procedure after calculating the equality factor of pure X (F_X_) at the two chosen wavelengths for Y.

Finally another novel method for simultaneous determination of quaternary mixtures was proposed and named double divisor-ratio difference-dual wave length (DD-RD-DW). It considered as one of the new applications of double divisor [19] and an extension to the double divisor-ratio difference method (DD-RD) [20] by coupling it with dual wavelength method.

For the determination of concentration of component of interest by the DD-RD-DW method, the component of interest shows a significant amplitude difference at two selected wavelengths λ_1_ and λ_2_ where the two interfering substances used as double divisor give constant amplitude as while the third one shows the same amplitude values at these two selected wavelengths.

This can be summarized in the following equations.

If we have a mixture of four drugs (X, Y, Z and W), dividing the spectrum of the quaternary mixture by the sum of the normalized spectra of Z and W (Z′ + W′) as a divisor, a constant value is generated in a certain region of wavelengths.

9 $${\text{Pm}} = \frac{{a_{X} C_{X} }}{{a_{Z} + a_{W} }}\; + \,\frac{{a_{Y} C_{Y} }}{{a_{Z} + a_{W} }}\; + \;{\text{constant}}$$

Suppose the amplitudes at the two selected wavelength are P_1_ and P_2_ at λ_1_ and λ_2_ (where Y has the same amplitude), respectively, then;

10 $${\text{P}}_{ 1} = \frac{{a_{X} C_{X} }}{{[a_{Z} + a_{W} ]1}} + \frac{{a_{Y} C_{Y} }}{{[a_{Z} + a_{W} ]1}} + {\text{constant}}$$

11 $${\text{P}}_{ 2} = \frac{{a_{X} C_{X} }}{{[a_{Z} + a_{W} ]2}} + \frac{{a_{Y} C_{Y} }}{{[a_{Z} + a_{W} ]2}} + {\text{constant}}$$

$$\frac{{a_{Y} C_{Y} }}{{[a_{Z}\,+\,a_{W} ]1}} = \frac{{a_{Y} C_{Y} }}{{[a_{Z}\,+\,a_{W} ]2}}$$

Then by subtraction

$${\text{P}}_{ 1} - {\text{P}}_{ 2} = \left( {\frac{{a_{X} C_{X} }}{{a_{Z + } a_{W} }}} \right)1 - \left( {\frac{{a_{X} C_{X} }}{{a_{Z + } a_{W} }}} \right)2$$

The concentration of X is calculated using the regression equation representing the linear correlation between the differences of ratio spectra amplitude at the two selected wavelengths to the corresponding concentrations of drug (X).

## Experimental

### Reagents and chemicals

- Pure samples—drotaverine (DRO) was kindly supplied by Alexandria Pharmaceuticals and Chemical Industries, Alexandria, Egypt. CAF and PCT were kindly supplied by Minapharm Pharmaceutical Company, Cairo, Egypt. Para-aminophenol was purchased from Sigma Aldrich, Germany. The purities were found to be 100.25 ± 0.39, 99.56 ± 0.59, 99.98 ± 0.25 and 99.99 ± 0.39 for DRO, CAF, PCT and PAP respectively.
- Market sample—*Petro* tablets, labelled to contain 40 mg (DRO)/400 mg (PCT)/60 mg (CAF), *Soumadril Compound* tablets labelled to contain 200 mg Carisopradol (CAR)/160 mg (PCT)/32 mg (CAF) and *Panadol Extra* tablets labelled to contain 500 mg (PCT)/65 mg (CAF), were purchased from the Egyptian market.
- Solvents—Spectroscopic analytical grade methanol (S.d.fine-chem limited-Mumbai).
- Stock standard solutions—(1 mg/mL) stock solution of each of DRO, CAF, PCT and PAP in methanol were prepared. The prepared solutions were found to be stable without any degradation when stored in the dark in the refrigerator at 4° C for 1 week except for PAP which should be freshly prepared.
- Working standard solutions—(50 μg/mL) working solutions for DRO, CAF, PCT and PAP were prepared from (1 mg/mL) stock solutions by appropriate dilutions with methanol.

### Apparatus

Spectrophotometric measurements were carried out on JASCO V-630 BIO Double-beam UV–Vis spectrophotometer (S/N C367961148), using 1.00 cm quartz cells. Scans were carried out in the range from 200 to 400 nm at 0.1 nm intervals. Spectra Manager II software was used.

### Procedures

#### Construction of calibration graphs

Aliquots equivalent to 10–260 μg DRO, 15–260 μg CAF, 10–240 μg PCT and 10–300 μg PAP were accurately transferred from their working standard solutions into four separate series of 10-mL volumetric flasks then completed to volume with the same solvent. The spectra of the prepared standard solutions were scanned from 200 to 400 nm and stored in the computer against methanol as a blank.

##### For DRO

A calibration graph was constructed relating the absorbance of zero order spectra (D^0^) of DRO at 228.5 nm versus the corresponding concentrations.

The stored (D^0^) spectra of DRO were divided by (a) the normalized spectrum of CAF, (b) the normalized spectrum of DRO, (c) sum of normalized spectrum of CAF and PAP, separately. Calibration graphs were constructed by plotting (a) the difference between the amplitudes at [263.6 and 291.8 nm], (b) the constant values measured from 310–400 nm, (c) the difference between the amplitudes at [315 and 336 nm] versus the corresponding DRO concentrations, respectively.

##### For CAF

Two calibration graphs were constructed using the zero order spectra (D^0^). The first one related the absorbance at 263.6 nm versus the corresponding CAF concentrations. While the second one related the difference between the absorbance at 231.5 and 263.6 nm versus the absorbance at 263.6 nm.

The (D^0^) spectra of CAF were divided by the normalized spectrum of PCT, and then two calibration graphs were constructed. The first was plotted between the amplitudes difference at [240 and 263.6 nm] versus amplitudes at 263.6 nm where as the second graph between the amplitudes difference at [233.8 and 273.7 nm] versus the corresponding CAF concentrations.

The stored (D^0^) spectra of CAF were also divided by the normalized spectrum of DRO and the obtained ratio spectra were manipulate for construction of another 2 calibration graphs. A graph was directly constructed between the amplitude difference at 265 and 295 nm multiplied by (5.58) versus the corresponding CAF concentrations and the regression equations were computed. The first derivative of the above ratio spectra was then recorded using scaling factor = 1 and ∆λ = 8 and a calibration graph between the amplitude at 219 nm versus the corresponding concentrations of CAF was constructed.

##### For PCT

A calibration graph was constructed relating the absorbance of zero order spectra (D^0^) of CAF or PCT at 263.6 nm versus the corresponding concentrations.

The stored (D^0^) spectra of PCT were divided by (a) the normalized spectrum of CAF, (b) normalized spectrum of DRO and (c) the sum of normalized spectrum of DRO and CAF, separately. Three calibration graphs were constructed by plotting (a) the amplitude differences between 219.2 and 252 nm, (b) amplitude differences between 257 and 230 nm multiplied by (4.73), (c) amplitude differences between 261.2 and 277.2 nm versus the corresponding PCT concentrations, respectively.

##### For PAP

The zero order spectra (D^0^) of PAP were scanned and manipulated to obtain two calibration graphs. Firstly, they were divided by the sum of normalized spectrum of DRO and CAF, to construct a calibration graph was constructed between the amplitude differences at 311 and 318 nm versus the corresponding PAP concentrations. Then their first derivative spectra (D^1^) were recorded using scaling factor = 10 and ∆λ = 8 and a calibration graph was constructed relating the amplitude of the obtained (D^1^) spectra of PAP at 314.5 nm versus the corresponding concentrations.

#### Application to laboratory prepared mixtures

Into a series of 10 mL volumetric flask, accurate aliquots of DRO, CAF, PCT and PAP were transferred from their working standard solutions to prepare five mixtures containing different ratios of the cited drugs. The volumes were completed with methanol.

Each drug in the quaternary mixture can be determined and analysed by more than one method using different approaches.

DRO was determined by four different methods; direct spectrophotometric method after derivative transformation, ratio difference-isosbestic points, induced ratio difference and double divisor-ratio difference-dual wavelength;

CAF was determined by five different methods; advanced absorbance subtraction, advanced amplitude modulation, simultaneous derivative ratio, ratio difference-isosbestic points and induced ratio difference.

PCT was determined using six different methods; advanced absorbance subtraction, advanced amplitude modulation, simultaneous derivative ratio, ratio difference-isosbestic points, induced ratio difference and double divisor-ratio difference-dual wavelength.

While PAP was determined adopting two methods; first derivative spectrophotometric method and double divisor-ratio difference-dual wavelength.

#### Application to pharmaceutical dosage form

Ten tablets of each of Petro^®^, Soumadril Compound^®^ and Panadol Extra^®^ formulations were accurately weighed, finely powdered and homogenously mixed. A portion of the powder equivalent to 5 mg PCT were separately weighed from Petro^®^ (A), Soumadril Compound^®^ (B) and Panadol Extra^®^ (C), respectively and dissolved in methanol by shaking in ultrasonic bath for about 30 min. The solution was filtered into a 100 mL measuring flask and the volume was completed with the same solvent. 2 mL were accurately transferred from the above prepared solutions of formulations (A, B) and 4 mL were accurately transferred from the solution of formulation (C), to three separate 10-mL volumetric flasks. The concentration of each drug was calculated using its specified methods. When carrying out the standard addition technique, different known concentrations of pure standard of each drug were added to the pharmaceutical dosage form before proceeding in the previously mentioned procedure.

## Results and discussion

**Fig. 2:**
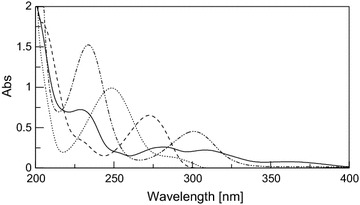
Zero order absorption spectra of 10 μg/mL DRO (*solid line*), 10 μg/mL PCT (*dotted line*), 10 μg/mL CAF (*dashed line*) and 10 μg/mL PAP (*dashed dotted line*)

By scanning the absorption spectra of DRO, CAF, PCT and PAP in the solution of dosage forms in methanol, severely overlapped spectral bands were observed in the wavelength region of 200–300 nm; which hindered their direct determination (Fig. 2). DRO showed extension over the PAP but with low absorptivity, in addition that PAP may exhibit a contribution at DRO extended region in high concentrations, and although PAP was more extended than CAF and PCT after 315 nm, but it can only be measured at a shoulder which could decrease sensitivity especially at high concentration of PCT which is the major component in all the proposed dosage forms.

**Fig. 3:**
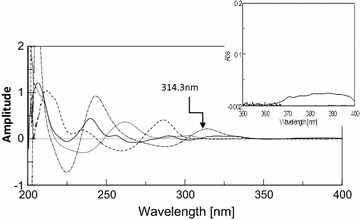
First order absorption spectra of 10 μg/mL DRO (*solid line*), 10 μg/mL PCT (*dotted line*), 10 μg/mL CAF (*dashed line*) and 10 μg/mL PAP (*dashed dotted line*)

**Fig. 4:**
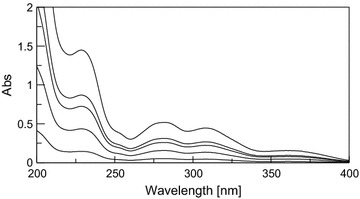
Zero order absorption spectra of DRO in mixtures (2, 6, 10, 12, and 20 μg/mL)

Upon derivatization using scaling factor = 10 and ∆λ = 8 nm, the contribution of PAP at the extended region of DRO was completely cancelled as shown in Fig. 3, but it was difficult to accurately measure the amplitude of DRO at its extended region due to its low absorptivity, so derivative transformation was adopted to overcome this problem. The derivative transformation was applied to obtain the (D^0^) of DRO by dividing the spectrum of the quaternary mixture by the first derivative of normalized spectrum of DRO (d/dλ) [a_DRO_], and then the constant generated in the region 360–380 nm was multiplied by the normalized spectrum of DRO [a_DRO_] where the absorbance of DRO can be measured at its 228.5 nm (λ_max_) giving maximum sensitivity and minimum error as shown in Fig. 4.

Also when the generated constant was multiplied by the first derivative of normalized spectrum of DRO used as divisor, the (D^1^) spectrum of DRO in the mixture was obtained and then subtracted from the total (D^1^) of the quaternary mixture via spectrum subtraction technique the spectrum of the first derivative of the resolved ternary mixture of CAF, PCT and PAP was obtained and PAP was determined by measuring the peak amplitude at 314.3 nm where CAF and PAP showed no contribution as shown in Fig. 3. Similarly, derivative transformation technique was adopted to obtain the D^0^ of PAP by dividing the spectrum of the above resolved ternary mixture by the first derivative of normalized spectrum of PAP (d/dλ) [a_PAP_], and then the constant generated in the region 310–330 nm was multiplied by the normalized spectrum of PAP [a_PAP_]. The obtained D^0^ of PAP was successively subtracted from the D^0^ spectrum of the resolved ternary mixture to get the D^0^ spectrum of binary mixture of CAF and PCT.

Three different novel, simple and accurate methods were adopted for simultaneous determination of CAF and PCT in presence of each other either in bulk, in different dosage forms as binary mixture and in presence of other components after their resolutions.

### Advanced absorbance subtraction

The absorption spectra of CAF and PCT are severely overlapped in the wavelength region of 200–300 nm and intersect at 3 isoabsorptive point 226.9, 263.6 and 292 nm where the mixture of the drugs acts as a single component and give the same absorbance value as pure drug.

The absorption spectra of the standard solutions of CAF with different concentrations were recorded in the wavelength range of 200–400 nm. Two wavelengths are selected (λ_iso_ of CAF 263.6 nm and λ_2_ = 231.5 nm) where PCT shows equal absorbance at these wavelengths. The absorbance difference ∆A (A_iso_ – A_231.5_) between two selected wavelengths on the mixture spectra is directly proportional to the concentration of CAF; while for PCT the absorbance difference inherently equals to zero. A calibration graph is constructed for pure CAF representing the relationship between (A_iso_ – A_2_) and A_iso_ and a regression equation was computed.

By substituting the absorbance difference ∆A (A_iso_ – A_2_) between the two selected wavelengths of the mixture spectrum in the above equation, the absorbance A_postulated_ corresponding to the absorbance of CAF only at A_iso_ was obtained.

Subtracting the postulated absorbance of CAF at A_iso_ from the practically recorded absorbance [A_Recorded_] at A_iso_ to get that corresponding to PCT.

The concentrations of CAF and PCT were calculated using the corresponding unified regression equation (obtained by plotting the absorbance of the zero order spectra of CAF or PCT at λ_iso_ 263.6 nm against the corresponding concentrations).

### Advanced amplitude modulation method (AAM)

**Fig. 5:**
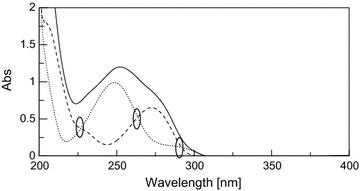
Zero order absorption spectra of 10 μg/mL PCT (*dotted line*) and CAF (*dashed line*) showing 3 isoabsorptive points at 226.9 263.6 and 292 nm and the binary mixture of CAF and PCT 10 μg/mL of each

**Fig. 6:**
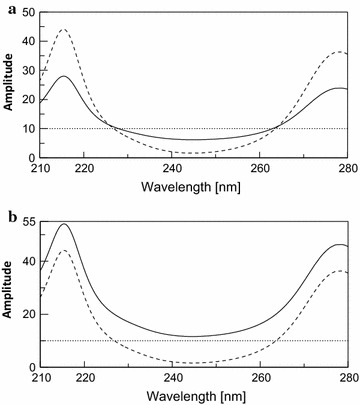
**a** Ratio absorption spectra of 10 μg/mL PCT (*dotted line*), 10 μg/mL CAF (*dashed line*) and the binary mixture of CAF and PCT 5 μg/mL of each (*dotted straight line*) obtained after division by the normalized spectra of PCT. **b** Ratio absorption spectra of 10 μg/mL PCT (*dotted line*), 10 μg/mL CAF (*dashed line*) and the binary mixture of CAF and PCT 10 μg/mL of each (*dotted straight line*) obtained after division by the normalized spectra of PCT

As shown in (Fig. 5), the absorption spectra of CAF and PCT in methanol shows isoabsorptive point at 263.6 nm (a_CAF_ = a_PCT_) which is retained at the same place in the ratio spectrum of CAF using the normalized spectrum of PCT as a divisor (Fig. 6a).

At first a regression equation was formulated representing the linear relationship between the amplitudes difference of different pure CAF concentrations at (263.6–240 nm) versus its corresponding amplitude 263.6 nm.

The AAM method was applied by dividing the spectrum of the binary mixture by the normalized divisor of PCT to obtain the ratio spectra (Fig. 6b). The amplitudes difference of the obtained ratio spectrum at 263.6 nm (λ_iso_) and 240 nm were recorded (∆Pm). And by substituting in the above regression equation previously formulated postulated amplitude of CAF alone at 263.6 nm (λ_iso_).

Subtracting the postulated amplitude of CAF at λ_iso_ from the practically recorded amplitude [P_Recorded_] of the binary mixture at λ_iso_ we get that corresponding to PCT.

The advantage of this method over the advanced absorbance subtraction method is the complete cancelling of the interfering component in the form of constant where the difference at any two points along its ratio spectrum will be equal to zero. So there is no need for critical selection of wavelengths which leads to highly reproducible and robust results.

### Simultaneous derivative ratio

Salinas et al. [21] developed derivative ratio spectrophotometry (^1^DD) method to remove the interference of one component and to determine the other. This method was then modulated to be simultaneous by coupling with amplitude modulation theory to generate simultaneous derivative ratio method (S^1^DD) [16]. In S^1^DD after division by the normalized spectra of PCT and before the derivatization step took place, the amplitude at isoabsorptive point (263.6 nm) was determined representing the actually concentration of CAF and/or PCT. Then derivative of these ratio spectra was obtained to remove the constant generated of PCT concentration in the division spectrum.

**Fig. 7:**
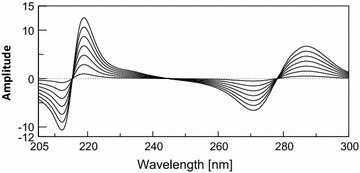
First derivative of ratio spectra of CAF (2–26 μg/mL) using normalized PCT spectrum as a divisor

Figure 7 shows the obtained derivative ratio spectra of different concentrations of CAF using scaling factor = 1 and ∆λ = 8 nm. A correlation between the peak amplitudes at 219 nm and the corresponding CAF concentration was plotted from which its concentration could be determined. The concentration of PCT was progressively determined by subtraction of the obtained CAF concentration from the total concentration at isosbestic point (λ_iso_ 263.6 nm) recorded before derivatization.

### For simultaneous determination of ternary mixture

#### Ratio difference-isosbestic points

**Fig. 8:**
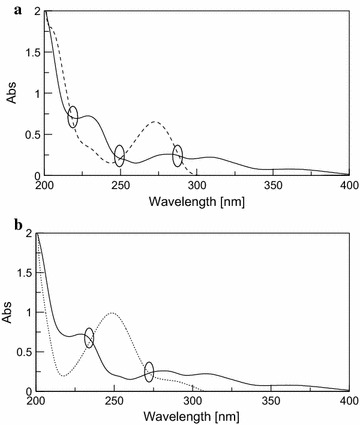
**a** Zero order absorption spectra of DRO (*solid line*) and CAF (*dashed line*) showing three isoabsorptive points at 219.2, 252 and 288 nm 10 μg/mL of each. **b** Zero order absorption spectra of DRO (*solid line*) and PCT (*dotted line*) showing two isoabsorptive points at 233.8 and 273.7 nm 10 μg/mL of each

The zero order of the studied drugs showed the presence of three isoabsorpative points between CAF and PCT as shown in Fig. 5, three isoabsorptive points are between DRO and CAF (Fig. 8a) while another two isoabsorptive points are between DRO and PCT (Fig. 8b).

**Fig. 9:**
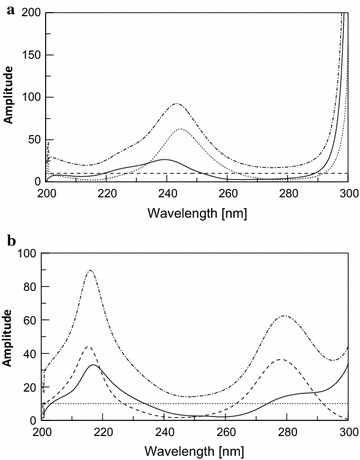
**a** Ratio spectra of DRO (*solid line*), CAF (*dashed line*), PCT (*dotted line*) and their ternary mixture (*dashed dotted line*) containing 10 μg/mL of each using normalized CAF spectrum as a divisor. **b** Ratio spectra of DRO (*solid line*), CAF (*dashed line*), PCT (*dotted line*) and their ternary mixture (*dashed dotted line*) containing 10 μg/mL of each using normalized PCT spectrum as a divisor

For determination of DRO the absorption spectrum of the mixture was divided by the absorption spectrum of the normalized spectra of CAF, the obtained ratio spectrum is shown in Fig. 9a.

Then the difference between the amplitudes at the two selected isosbestic points between CAF and PCT (263.6 and 291.8 nm) was directly proportional to DRO concentration only.

For determination of PCT, the difference between the amplitude of the above ratio spectra obtained after dividing the spectrum of the ternary mixture by the normalized spectrum of CAF at the two selected isosbestic points (219.2 and 252 nm) between CAF and DRO was corresponding to PCT concentration only as shown in Fig. 9a.

The same procedures were applied for determination of CAF where the absorption spectrum of the mixture was divided by the absorption spectrum of the normalized spectra of PCT as divisor and the difference between the amplitude at the two selected isosbestic points (233.8 and 273.7 nm) between DRO and PCT was corresponding to CAF concentration only as shown in Fig. 9b.

#### Induced ratio difference method

**Fig. 10:**
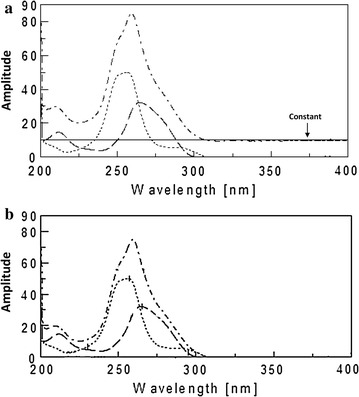
Ratio spectra of DRO (*solid line*), CAF (*dashed line*), PCT (*dotted line*) and their ternary mixture (*dashed dotted line*) containing 10 μg/mL of each using normalized DRO spectrum as a divisor. **b** Ratio spectra of CAF (*dashed line*), PCT (*dotted line*) and their resolved binary mixture (*dashed dotted line*) containing 10 μg/mL of each using normalized DRO spectrum as a divisor after subtraction of the obtained constant

The concentration of DRO was determined using amplitude modulation method from the straight line parallel to the x-axis in the extended region at 310–400 nm for DRO as shown in Fig. 10a. The obtained constants of DRO are then subtracted from the total ratio spectra of the mixture obtaining the ratio spectra of binary mixtures of both CAF and PCT divided by normalized spectra of DRO as shown in Fig. 10b.

By screening the ratio spectra of pure CAF divided by the normalized spectra of DRO, two wavelengths were selected, 265 and 295 nm, where 265 nm showed the maximum peak in order to obtain maximum sensitivity. To cancel the contribution of PCT at both selected wavelengths, induced dual wave length method was adopted by calculating an equality factor for pure PCT at two selected wave lengths of CAF $\left( {{\text{F}} \; = \; \left[ {{\text{P}}_{ 2 6 5} /{\text{P}}_{ 2 9 5} } \right] \; = \; 5. 5 8} \right)$ as shown in Fig. 10b.

In order to determine of PCT, the same procedures were applied as described for CAF. The two selected wavelengths were 257 nm (maximum peak amplitude) and 230 nm. The factor that equalize the amplitude of CAF at the selected wavelengths was calculated $\left( {{\text{F}}\, = \;\left[ {{\text{P}}_{ 2 5 7} /{\text{P}}_{ 2 30} } \right] \; = \; 4. 7 3} \right)$.

### For simultaneous determination of quaternary mixture

#### Double divisor-ratio difference-dual wave length

For the successful application of the proposed method, it is a must to obtain a constant region in the ratio spectra resulted after dividing the total spectrum of any two drugs by the sum of their normalized spectra.

**Fig. 11:**
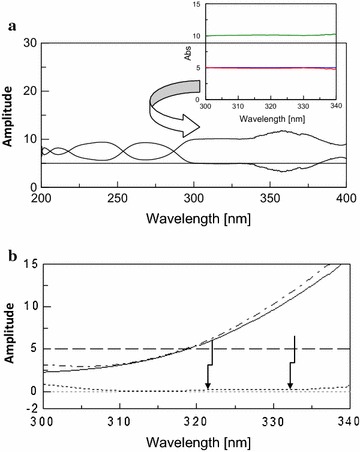
**a** Ratio spectra of three binary mixtures of CAF and PAP in different concentrations using the sum of normalized spectra of CAF and PAP as double divisor showing the obtained constant region. **b** Ratio spectra of DRO (*solid line*), binary mixture of CAF and PAP (*dashed line*), PCT (*dotted line*) and their quaternary mixture (*dashed dotted line*), 5 μg/mL each using the sum of normalized spectra of CAF and PAP as double divisor

For determination of DRO, the spectra of quaternary mixtures of DRO, CAF, PCT and PAP were divided by the sum of the normalized spectra of both CAF and PAP where a constant region from 300–340 nm was generated for CAF and PAP as shown in Fig. 11a. A correlation was obtained between the amplitude difference at 315 and 336 nm at which PCT have the same amplitude $\left( {\Delta {\text{P}}_{\text{PCT}} = {\text{P}}_{ 1} - {\text{P}}_{ 2} = {\text{zero}}} \right)$ and the corresponding DRO concentration was plotted from which its concentration could be determined as shown in Fig. 11b.

**Fig. 12:**
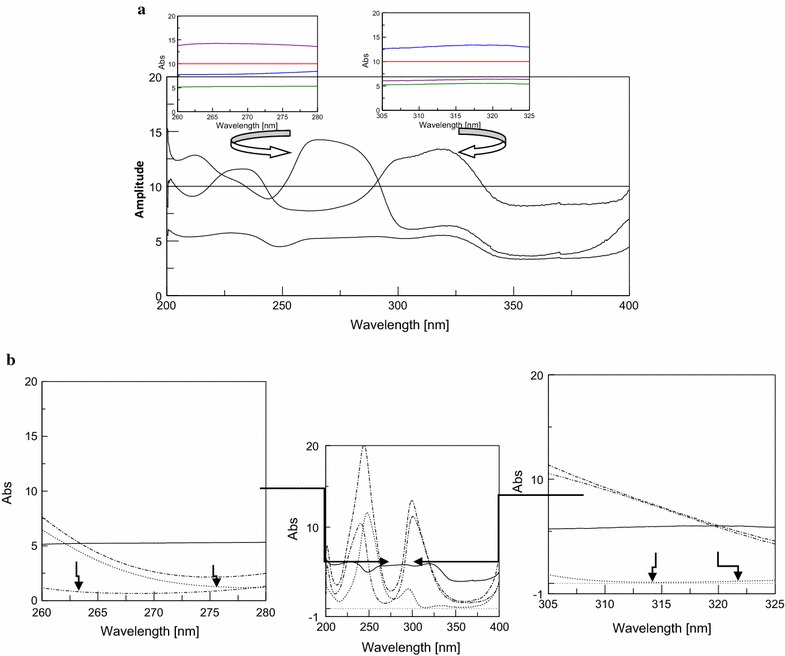
**a** Ratio spectra of 4 binary mixtures of DRO and CAF in different concentrations using the sum of normalized spectra of DRO and CAF as double divisor showing the obtained constant regions. **b** Ratio spectra of binary mixture of DRO and CAF (*solid line*), PCT (*dotted line*), PAP (*dashed single dotted line*) and their quaternary mixture (*dashed double dotted line*), 5 μg/mL each using the sum of normalized spectra of CAF and PAP as double divisor

For determination of PCT and PAP, the spectra of quaternary mixtures were divided by the sum of normalized spectra of both DRO and CAF, where constant regions at 260–280 nm and at 307–325 nm for DRO and CAF were obtained as shown in Fig. 12a. A correlation was obtained between the amplitude difference at 261.2 and 277.2 nm at which PAP have the same amplitude $\left( {\Delta {\text{P}}_{\text{PAP}} = {\text{P}}_{ 1} - {\text{P}}_{ 2} = {\text{zero}}} \right)$ and the corresponding PCT concentration was plotted from which its concentration could be determined as shown in Fig. 12b. While for PAP the correlation was obtained between the amplitude difference at 311 and 318 nm at which PCT have the same amplitude $\left( {\Delta {\text{P}}_{\text{PCT}} = {\text{P}}_{ 1} - {\text{P}}_{ 2} = {\text{zero}}} \right)$ and the corresponding PAP concentration was plotted from which its concentration could be determined as shown in Fig. 12b.

The method failed in determination of CAF. The main disadvantage of this method is the restriction in the choice of the selected wavelengths which are restricted to those wavelengths with constant absorbance of the interfering substance.

The proposed spectrophotometric methods were compared to a recently reported HPLC method [10] in which a separation was achieved on a C_18_ column (250 mm × 4.6 mm, 5 μm particle size), using methanol and 0.02 M phosphate buffer, pH 4.0 (50:50, v/v) as a mobile phase and UV detection at 220 nm. The chromatographic method showed better sensitivity where concentrations up to 0.5 µg/mL of each of DRO, CAF and PCT could be quantified. While the proposed novel spectrophotometric methods showed wider range. In addition the presented methods were capable to determine the concentration of PAP which is the main degradation products and synthetic impurity of PCT and thus could be considered as stability indicating methods. Also it needs no tedious conditions optimization as that required for the chromatographic method. The proposed spectrophotometric methods are also considered to be fast and time saving where the analysis of the quaternary or the ternary mixture takes few seconds once calibration graphs were constructed and regression equations are computed where all the reported chromatographic techniques needs at least 10 min in a single run to resolve the ternary mixture.

## Method validation

The proposed spectrophotometric methods were validated in compliance with the ICH guidelines [22], as shown in Table 1.

**Table 1:** Assay parameters and method validation obtained by applying the proposed spectrophotometric methods for determination of DRO, CAF, PCT and PAP

Parameters	DRO				CAF				
	D^0^	RD-Iso	IRD	DD-RD-DW	AAS	AAM	S^1^DD	RD-Iso	IRD
Linearity (µg/mL)	1–26				1.5–26				
Slope	0.0722	1.4648	1.0018	0.3179	0.0525	0.9961	0.5027	2.7068	1.92333
Intercept	0.0036	0.0727	0.0036	0.0158	−0.0005	0.0091	−0.0008	−0.0043	−0.003
Correlation coefficient (r)	1.0000	1.0000	1.0000	0.9999	1.0000	1.0000	1.0000	1.0000	1.0000
Accuracy	99.74 ± 0.40	99.67 ± 0.41	100.04 ± 0.29	100.77 ± 0.37	100.05 ± 0.14	100.25 ± 0.02	99.99 ± 0.01	99.96 ± 0.02	100.08 ± 0.02
RSD%^a^	0.249	0.248	0.105	0.398	0.110	0.115	0.187	0.177	0.110
RSD%^b^	0.317	0.315	0.186	0.372	0.205	0.207	0.223	0.231	0.211

Parameters	PCT						PAP		
	AAS	AAM	S^1^DD	RD-Iso	IRD	DD-RD-DW	D^1^	DD-RD-DW	
Linearity (µg/mL)	1–24	1–30							
Slope	0.0525	0.9961	0.9961	3.6059	1.9062	0.945	0.0213	1.8265	
Intercept	0.0005	0.0091	0.0091	0.0331	0.0175	0.0087	0.0010	0.0886	
Correlation coefficient (r)	1.0000	1.0000	1.0000	1.0000	1.0000	0.9999	0.9999	0.9999	
Accuracy	100.15 ± 0.31	100.30 ± 0.20	99.97 ± 0.25	100.01 ± 0.17	100.20 ± 0.21	99.98 ± 0.19	101.81 ± 0.40	100.146 ± 0.39	
RSD%^a^	0.380	0.315	0.382	0.279	0.201	0.374	0.727	0.732	
RSD%^b^	0.388	0.372	0.324	0.286	0.228	0.293	0.857	0.860	

RSD%^a^, RSD%^b^: the intra-day, inter-day respectively (n = 3) relative standard deviation of concentrations DRO (6, 14, 22 µg/mL), CAF (6, 14, 22 µg/mL), PCT (6, 14, 22 µg/mL) and PAP (10, 18, 26 µg/mL)

The specificity of the proposed methods was assessed by the analysis of laboratory prepared mixtures containing different ratios of the drugs, where satisfactory results were obtained over the calibration range as shown in Table 2. The proposed methods were also applied for the determination of the drugs in Petro, Soumadril Compound and Panadol Extra tablets. The validity of the proposed methods was further assessed by applying the standard addition technique as presented in Table 3. In Soumadril Compound, Carisopradol which is an open aliphatic structure doesn’t show any interference, therefore the mixture acts as a binary mixture of CAF and PCT.

**Table 2:** Determination of DRO, CAF, and PCT and PAP in laboratory prepared mixtures and pharmaceutical dosage forms by the proposed methods and results obtained by standard addition technique

Concentration (µg/mL)				Recovery%^a^								
Lab prepared mix.				DRO				CAF				
DRO	CAF	PCT	PAP	D^0^	RD-Iso	IRD	DD-RD-DW	AAS	AAM	S^1^DD	RD-Iso	IRD
2^b^	3	20	2	101.24	98.78	100.95	101.25	99.46	100.35	100.27	100.22	100.27
10	10	10	1	98.24	99.75	99.33	98.24	99.65	100.17	99.98	99.97	99.98
6	12	6	6	99.42	99.41	99.87	99.42	99.94	100.43	100.23	100.22	100.24
20	8	4	4	100.13	100.05	99.96	100.14	99.62	100.18	100.00	99.98	100.01
12	20	8	2	99.83	99.82	99.84	99.84	99.78	100.22	100.00	100.01	100.00
0^c^	2	10	1					99.97	99.97	100.19	101.20	100.15
0^d^	2.6	20	2					100.07	99.89	100.21	99.67	100.20
Mean ± SD				99.77 ± 1.09	99.56 ± 0.49	99.99 ± 0.53	99.78 ± 1.09	99.78 ± 0.21	100.17 ± 0.19	100.12 ±0.12	100.20 ± 0.52	100.12 ± 0.12

Concentration (µg/mL)				Recovery%^a^							
Lab prepared mix.				PCT						PAP	
DRO	CAF	PCT	PAP	AAS	AAM	S^1^DD	RD-Iso	IRD	DD-RD-DW	D^1^	DD-RD-DW
2^b^	3	20	2	100.11	101.21	99.80	100.21	100.12	100.12	99.20	99.85
10	10	10	1	100.07	100.65	100.02	100.26	100.07	100.08	98.66	99.61
6	12	6	6	100.33	100.92	100.59	100.65	100.34	100.34	102.38	100.68
20	8	4	4	99.84	100.37	100.25	100.39	99.94	99.94	98.80	98.80
12	20	8	2	100.18	99.98	100.46	100.35	100.12	100.12	99.65	98.00
0^c^	2	10	1	100.21	100.89	100.45	99.65	100.31	99.89	99.42	99.38
0^d^	2.6	20	2	99.95	99.91	99.97	99.82	99.76	100.32	98.02	100.75
Mean ± SD				100.10 ± 0.16	100.56 ± 0.49	100.22 ± 0.29	100.19 ± 0.34	100.09 ± 0.20	100.11 ± 0.17	99.45 ± 1.40	99.57 ± 0.99

^a^Average of three determinations

^b^The ratio of the lab mixture in Petro tablets

^c^The ratio of the lab mixture of (CAF:PCT) in Soumadril Compound tablets

^d^The ratio of the lab mixture of (CAF:PCT) in Panadol Extra tablets

**Table 3:** Determination of DRO, CAF, and PCT and PAP in pharmaceutical dosage forms by the proposed methods and results obtained by standard addition technique

Pharmaceutical dosage form	Recovery%^a^														
	DRO				CAF					PCT					
	D^0^	RD-Iso	IRD	DD-RD-DW	AAS	AAM	S^1^DD	RD-Iso	IRD	AAS	AAM	S^1^DD	RD-Iso	IRD	DD-RD-DW
Petro 40(DRO)/400(PCT)/60 (CAF), mean ± SD	99.79 ± 0.48	99.93 ± 0.57	99.91 ± 0.30	99.80 ± 0.49	99.01 ± 0.81	99.90 ± 0.78	99.82 ± 0.65	99.72 ± 0.79	99.83 ± 0.70	100.14 ± 0.21	100.18 ± 0.58	99.63 ± 0.40	100.08 ± 0.45	100.19 ± 0.16	100.05 ± 0.27
Standard addition, mean ± SD	99.63 ± 0.22	99.70 ± 0.35	99.27 ± 0.16	99.57 ± 0.44	101.61 ± 0.97	100.98 ± 0.35	100.32 ± 0.21	99.51 ± 1.80	98.98 ± 1.66	99.96 ± 1.71	99.85 ± 0.05	100.78 ± 1.30	100.84 ± 0.64	100.58 ± 0.59	100.59 ± 0.72
Soumadril Comp. 200 (CAR)/160 (PCT)/32 (CAF), mean ± SD					100.76 ± 0.49	100.02 ± 0.51	100.01 ± 0.51	99.86 ± 0.46	100.01 ± 0.41	100.20 ± 0.62	99.52 ± 0.43	100.58 ± 0.61	101.03 ± 0.53	100.85 ± 0.47	100.75 ± 0.62
Standard addition, mean ± SD					101.08 ± 0.76	101.23 ± 0.43	100.88 ± 0.59	99.63 ± 0.85	99.57 ± 1.21	100.48 ± 1.35	99.30 ± 0.32	100.64 ± 0.60	100.77 ± 0.55	100. 81 ± 0.68	100.47 ± 0.85
Panadol Extra 500 (PCT)/65 (CAF), mean ± SD					100.55 ± 0.36	100.05 ± 0.39	99.76 ± 0.78	99.68 ± 0.73	100.08 ± 0.31	100.14 ± 0.58	100.01 ± 0.82	100.22 ± 0.30	100.27 ± 0.28	100.32 ± 0.71	100.54 ± 0.25
Standard addition, mean ± SD					100.38 ± 0.55	100.79 ± 0.84	100.54 ± 0.57	99.88 ± 0.47	100.17 ± 1.35	100.24 ± 0.82	99.57 ± 0.54	99.86 ± 0.57	100.34 ± 0.68	100. 08 ± 0.78	100.29 ± 0.61

^a^Average of three determinations

## Statistical analysis

Table 4 showed statistical comparisons of the results obtained by the proposed methods and reported method for DRO [23], and official methods for CAF [24] and PCT [25]. The calculated *t* and F values were less than the theoretical ones indicating that there was no significant difference between them with respect to accuracy and precision.

**Table 4:** Statistical comparison for the results obtained by the proposed spectrophotometric methods, the reported method [23] for the analysis of DRO and official methods [24, 25] for analysis of CAF and PCT

Values	DRO					CAF						PCT						
	Proposed methods				Reported method [23]^a^	Proposed methods					Official method [24]^b^	Proposed methods						Official method [25]^c^
	D^0^	RD-Iso	IRD	DD-RD-DW		AAS	AAM	S^1^DD	RD-Iso	IRD		AAS	AAM	S^1^DD	RD-Iso	IRD	DD-RD-DW	
Mean	99.79	99.93	99.91	99.80	100.25	99.01	99.90	99.82	99.72	99.83	99.56	100.14	100.18	99.63	100.08	100.19	100.05	99.98
SD	0.48	0.57	0.30	0.49	0.39	0.81	0.78	0.65	0.79	0.70	0.59	0.21	0.58	0.40	0.45	0.16	0.27	0.25
RSD%	0.481	0.570	0.300	0.490	0.389	0.818	0.780	0.651	0.792	0.701	0.592	2.097	0.578	0.402	0.449	0.159	0.269	0.250
n	5	5	5	5	5	5	5	5	5	5	5	5	5	5	5	5	5	5
Variance	0.2304	0.324	0.0900	0.240	0.1521	0.6561	0.6084	0.4225	0.6241	0.49	0.3481	0.0441	0.3364	0.1600	0.2025	0.0256	0.0729	0.0625
Student’s t test^d^ (2.306)	1.663	1.036	0.1609	1.607		1.227	0.7774	0.6623	0.3629	0.5281		1.096	0.7081	1.659	0.4344	0.1523	0.4254	
F value^d^	1.515	2.136	1.690	1.579		1.885	1.748	1.214	1.793	1.408		1.417	5.382	2.560	3.240	2.441	1.166	

^a^Dualwavelength spectrophotometric method at 271.5 and 280.0 nm

^b^Potentiometric titration using 0.1 M perchloric acid

^c^Direct UV spectrophotometric method, measuring the absorbance in water at 244 nm

^d^The values in the parenthesis are the corresponding theoretical values of *t* and *F* at *P* = 0.05

## Conclusions

In this work more than eight novel and smart spectrophotometric methods were developed and validated for the resolution of the quaternary mixtures either successively or progressively. Drotaverine, caffeine, paracetamol and para-aminophenol, the main degradation product and synthetic impurity of Paracetamol quaternary mixture was taken as a model for application of the proposed methods.

It could be concluded that the proposed procedures are accurate, simple and reproducible and yet economic. They are also sensitive and selective and could be used for routine analysis of complex most of the binary, ternary and quaternary mixtures and even more complex mixtures. The proposed methods also showed the significance of isoabsorptive point, normalized spectra as divisors and dual wavelengths as powerful tools that could either be used alone or in combination with each other for the resolution of severely overlapped spectra without preliminary separation.
